# The Effect of Different Types of Physical Exercise on the Behavioural and Physiological Parameters of Standardbred Horses Housed in Single Stalls

**DOI:** 10.1155/2014/875051

**Published:** 2014-01-22

**Authors:** Barbara Padalino, Paola Zaccagnino, Pietro Celi

**Affiliations:** ^1^Department of Veterinary Medicine, University of Bari, Str. prov. Per Casamassima, km 3, 70010 Valenzano (Bari), Italy; ^2^Dipartimento di Scienze delle Produzioni Animali, Università degli Studi della Basilicata, Via dell'Ateneo Lucano 10, 85100 Potenza, Italy; ^3^Faculty of Veterinary Science, University of Sydney, P.M.B. 4003, Narellan, NSW 2567, Australia; ^4^Melbourne School of Land and Environment, The University of Melbourne, Parkville, VIC 3010, Australia

## Abstract

The aim of this study was to investigate the impacts of three different physical exercises on the physiological and behavioural patterns of Standardbred trotters housed in single stalls. Twelve racing mares were observed twice during each different exercise: daily training (DT) consisted of forty minutes at slow trot (4-5 m/s) in a small track; maximal exercise (ME) consisted of 1600 m run at maximal velocity; race (R) was a real race of 1600 m. The mares were examined at rest in their stall (Time I), soon after the completion of the exercise (Time II), one hour (Time III), and two hours (Time IV) after the exercise. Their heart rate, respiratory rate, and rectal temperature were recorded and they were videotaped in order to complete a focal animal sampling ethogram. All physiological parameters increased after exercise, in accordance with its intensity. After R and ME horses spent more time drinking, eating, and standing. The incidence of abnormal behaviours was very low and it was not affected by the different types of exercise. Overall, the assessment of horse behaviour after physical exercise by means of a focal animal sampling ethogram represents a useful tool to monitor equine welfare.

## 1. Introduction

Although animal welfare has become more important in the equine industry, housing systems limiting natural behaviour patterns are still widespread [1]. Single stalls can confine social interaction and locomotion to a great extent, but, despite this, they are widely adopted in the equine industry, especially for Standardbred horses [2]. Housing horses in a single stall can influence equine welfare [3], with physical exercise being the only moment when horses can regularly move and express social behaviour. However, the possible consequences of prolonged confinement in stables on equine well-being have been studied [4] and group housing systems have been tested [5]. Animals can adapt to a new environment, but, when horses are kept alone on a flat, unstimulating square with nothing to do, some adaptative responses might include apathy and unresponsiveness, hyperresponsiveness, and stereotypic behaviour [6]. Apparently functionless, repetitive, stereotypic activities can be seen in stable horses [7–9] and equine stereotypies are primarily based on feeding and locomotory behaviours, due to management practices that limit foraging behaviour and social contact [10].

Although some researchers are investigating the relationships between exercise, housing and management, and the development of abnormal behaviour in horses [11], there is a lack of information about the effect of traditional training and racing on the Standardbred horse's behaviour. By understanding the behaviour modification that different kinds of exercise could induce, the management of racing horses could be improved especially in terms of their welfare status. Therefore, we sought to evaluate the effect of two different types of exercise, as well as racing, on some behavioural patterns of Standardbred horses kept in single boxes. Some physiological parameters, namely, heart rate (HR), respiratory rate (RR), and temperature (*T*), were also monitored.

## 2. Materials and Methods

### 2.1. Animals, Management, and Physical Exercises

Twelve Standardbred mares, homogeneous for live weight (450 ± 25 kg), body condition score (3 ± 0.25 arbitrary units; from 1 to 5 accordingly with Martin-Rosset [12]), and age (3.5 ± 0.5 years), were recruited in this study. All mares were in good health status and they all were qualified to race (mean record on 1600 m race track was 1′  16′′ ± 02′′). The research was carried out on a racetrack in Castelluccio dei Sauri, Foggia (Italy), during the summer months of June and July where the average maximum and minimum temperature and relative humidity were 31 ± 4°C, 16 ± 3°C, and 61 ± 5%, respectively. All mares were housed at the racetrack in a stable which contained 16 single stalls (3.00 m ∗ 3.50 m, 10.5 m^2^) in two rows, with a central aisle (3 m wide). The front has sliding doors, divided in two parts with the bottom panel being always kept shut and the top one always open, when the mares were monitored for behavioural status.

The horses were fed with hay and concentrate three times a day: early in the morning (7 am), after the completion of the physical exercises (between 12 pm and 1 pm), and late in the afternoon (6 pm). The amount of feed, feed quality and type remained constant over the course of the experiment. Water was available in the stable at all times for each individual horse.

All mares were housed in the same horse stalls, trained and raced by the same trainer, and performed the following physical exercises at the same time of day during the whole study. (1) Daily training (DT): consisted of forty minutes at a slow trot (4-5 m/s) on the small track of the race track; mares would cover an average of 10,000 m. The mares were jugged to a heavy gig by Custom [13]. (2) Maximal exercise (ME): consisted of 1600 m at maximal velocity; the mares were jugged to a sulky and they were alone on the track. Before performing the ME, mares were warmed up with a 5,000 m trot at the speed of 6-7 m/s. (3) Race (R): consisted of a real race over a 1600 m distance. All races were in the morning from 11 am to 1 pm (matinee' race); DT and ME were also performed at a similar time of the day. The number of the horses in each race was about 14. Before the race, horses were warmed up with a 5000 m trot at the speed of 6-7 m/s. Immediately after each exercise the mares were showered, walked by an operator for 5 minutes, and then returned to their individual stalls. The weekly training regime consisted of three days of DT followed by one day of ME and then three days of DT again. Horses raced every fortnight and they rested the day after the race in paddocks. During DT and ME, horses performed the physical exercises in an empty racetrack, while during the race the racetrack had several spectators.

### 2.2. Behavioural and Physiological Parameters

The mares were examined at rest in their stalls (Time I) at 8 AM, within 10 ± 2 minutes after exercise, before washing and cooling down (Time II), one hour after exercise (Time III), and two hours after exercise (Time IV). Each examination included recording the heart rate (HR) by auscultation, respiratory rate (RR) through observation of chest-wall movement, and rectal temperature (RT) with an electronic thermometer in °C (Vedodigit II-PIC). All measures were taken by the same veterinarian. The mares were videotaped by a video camera (Sony) while they were in their stalls. Briefly, each observation cycle was 1 hour in duration for a total period of 4 hours, with one hour of observation before exercise and three hours observation after exercise: the first took place from 8 to 9 AM, before the training session (Time I), the second was when the horse came back to the stall after exercise (Time II), the third was one hour later (Time III), and the last one was two hours later (Time IV). Between the three observation periods after exercise there was a 5-minute break during which the physiological parameters described above were taken. One operator reviewed all the videos and compiled a “focal animal sampling ethogram” and the duration (sec) of the following behavioural states was calculated: lying, standing, drinking, hay and concentrate feeding, and walking, eliminative and explorative behaviours. Standing was defined as the time spent in station without doing other activities, such as feeding or drinking, while explorative behaviour was defined as the time that the mares spent sniffing each part of the box. Moreover, the duration of the following events was also calculated: urination, defecation, hay and concentrate feeding, and stereotypical and abnormal behaviour. Particular attention was placed on the possible identification of the following abnormal behaviours reported for Standardbreds: weaving, box-walking, crib-biting/wind sucking, and wood chewing [7]. All mares were observed twice for each exercise.

### 2.3. Ethical Guidelines

All the procedures were carried out in accordance with the Italian legislation on animal care (DL n. 116, 27/01/1992).

#### 2.3.1. Statistical Analysis

All data were normally distributed and were analysed by using REML variance component analysis procedure for *GenStat version 14,* where the type of physical exercise (R, ME, and DT), the time of observation (Times I, II, III, and IV), and the interaction between physical exercise and time of observation were considered as fixed factors, while mares and replicate were considered as random factors. All data were expressed as mean ± SE. The effects were considered to be significant at *P* < 0.05; differences between means were tested using least significant difference.

## 3. Results

**Figure 1:**
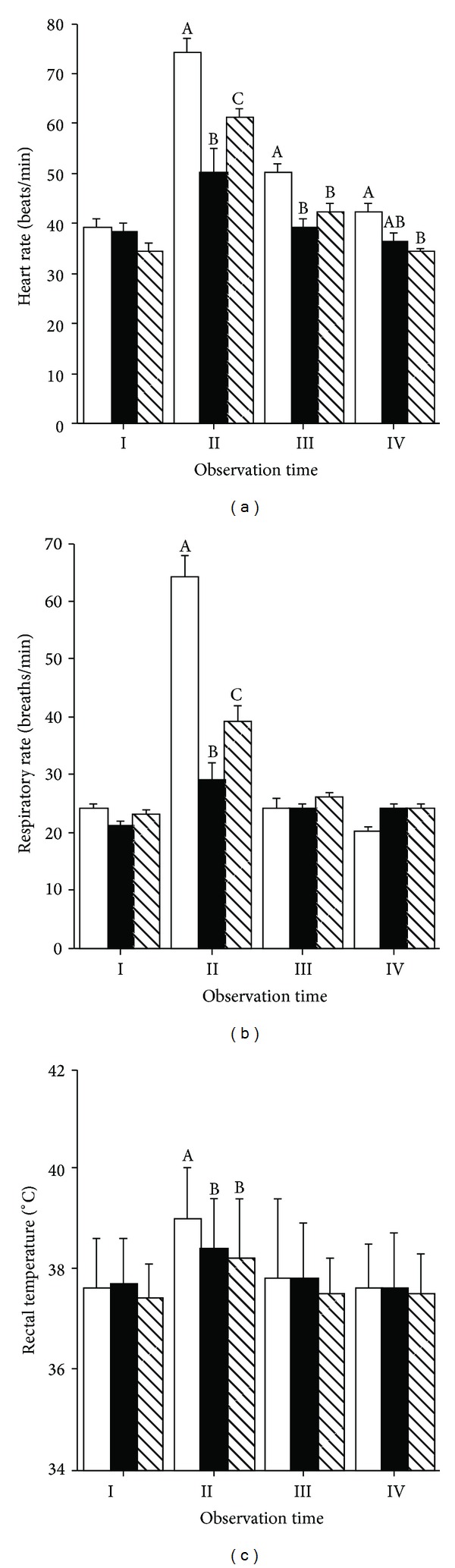
Effect of different types of physical exercise (race blank; maximal exercise shaded; daily exercise lined) on heart rate (a), respiratory rate (b), and rectal temperature (c) of Standardbred horses housed in single stalls. Mares were examined at rest in their stalls (Time I), immediately after exercise (Time II), one (Time III), and two hours (Time V) after the completion of exercise. Values are expressed as means ± SE. For parameters where a significant effect of type of exercise × time interaction was noted, means with different capital letters indicate significant difference (*P* < 0.01) between types of exercises.

A significant effect of physical exercise (*P* < 0.001), time (*P* < 0.001), and their interaction (*P* < 0.001) was observed for both heart rate (HR) (Figure 1(a)) and respiratory rate (RR) (Figure 1(b)). Both HR and RR were similar before the commencement of physical exercise and as expected they increased significantly soon after the completion of physical exercise (Time II) and then returned to preexercise levels by the end of the observation period (Time IV). A significant effect of the interaction time of observation × physical exercise was noted on both HR and RR (*P* < 0.001), with horses that undertook the race (R) presenting higher HR and RR levels than the horse that performed the daily training (DT) or maximal velocity (ME) exercises at Time II. HR levels were still higher in horses that performed the R exercise compared to those that performed the DT and ME exercises at Time III; at Time IV, horses that performed the R exercise had higher HR values than horses that performed the DT exercise. Differences in RR values between the race and the other two exercises disappeared after 1 h (Time III) from the completion of physical exercise. A significant effect of physical exercise (*P* < 0.001), time (*P* < 0.001), and their interaction (*P* < 0.05) was observed for rectal temperature (RT) with its values increasing soon after completion of the three different physical exercises (Time II) (Figure 1(c)). This increase was more pronounced in horses that performed the race. RT levels returned to preexercise levels 1 h (Time III) from the completion of physical exercise.

**Figure 2:**
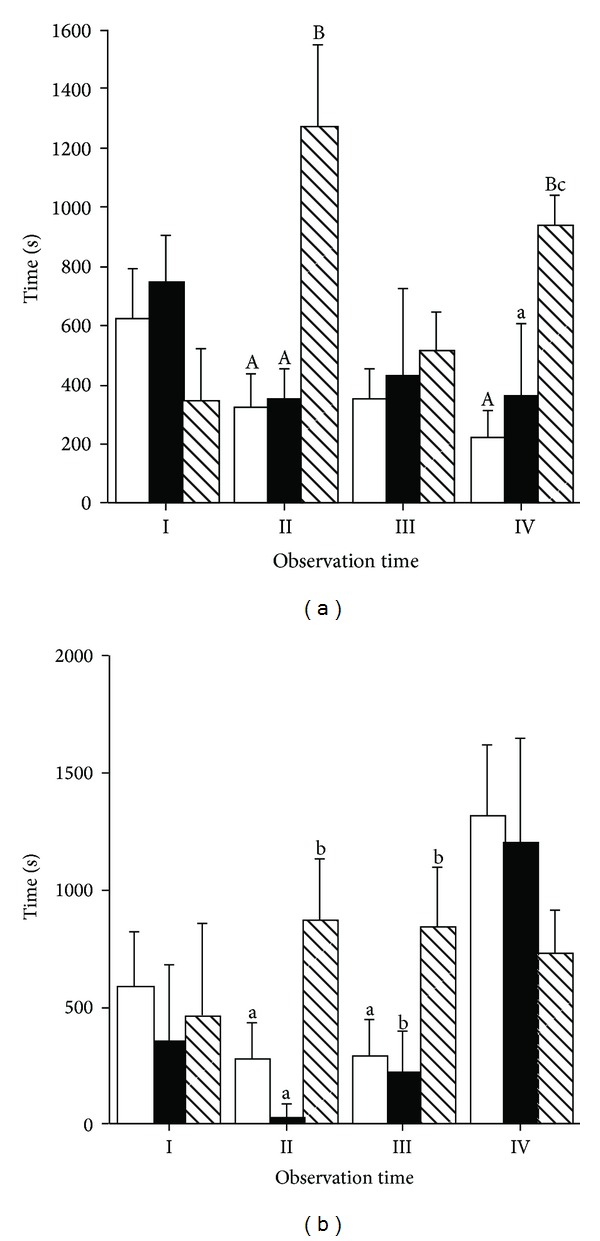
Effect of different types of physical exercise (race blank; maximal exercise shaded; daily exercise lined) on standing up activity (a) and resting behaviour (b) of Standardbred horses housed in single stalls. Mares were examined at rest in their stalls (Time I), immediately after exercise (Time II), one (Time III), and two hours (Time V) after the completion of exercise. Values are expressed as means ± SE. For parameters where a significant effect of type of exercise × time interaction was noted, means with different capital and low letters indicate significant difference (*P* < 0.01 and *P* < 0.05, resp.) between types of exercises.

A significant effect of physical exercise (*P* < 0.05) and physical exercise × time interaction (*P* < 0.01) was observed on standing activity (Figure 2(a)). Standing behaviour was significantly lower in horses that performed the R and ME exercises than in horses that performed the DT exercise on Times II and IV. A significant effect of time (*P* < 0.01) and physical exercise × time interaction (*P* < 0.05) was observed on resting behaviour (lying) (Figure 2(b)). Resting behaviour was significantly lower in horses that performed the R and ME exercises than in horses that performed the DT exercise on Times II and III.

**Figure 3:**
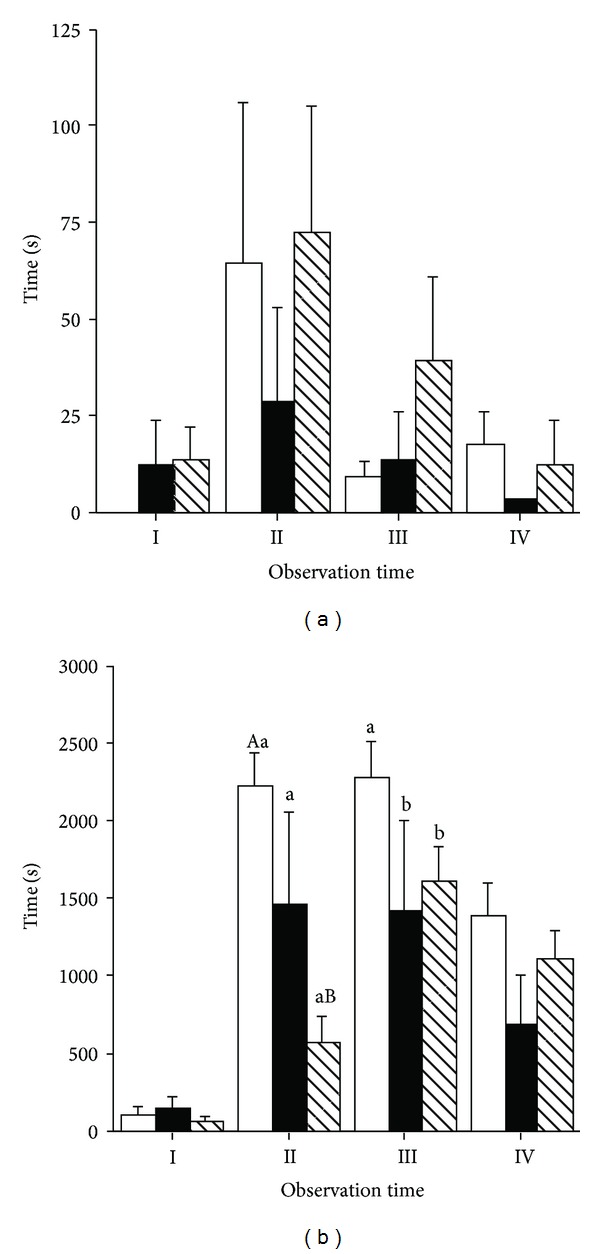
Effect of different types of physical exercise (race blank; maximal exercise shaded; daily exercise lined) on drinking (a) and eating activity (b) of Standardbred horses housed in single stalls. Mares were examined at rest in their stalls (Time I), immediately after exercise (Time II), one (Time III), and two hours (Time V) after the completion of exercise. Values are expressed as means ± SE. For parameters where a significant effect of type of exercise × time interaction was noted, means with different capital and low letters indicate significant difference (*P* < 0.01 and *P* < 0.05, resp.) between types of exercises.

A significant effect of time (*P* < 0.001) was observed for drinking activity with horses spending a higher amount of time drinking soon after the completion of physical exercise (Figure 3(a)). A significant effect of physical exercise (*P* < 0.05), time (*P* < 0.001), and their interaction (*P* < 0.05) was noted for eating activity (Figure 3(b)). One hour after the completion of physical exercises (Time II), horses that undertook the R and ME exercises spent more time eating than horses that performed the DT exercise. On Time III horses that undertook the R exercise spent more time eating than horses that performed the DT and ME exercises.

**Figure 4:**
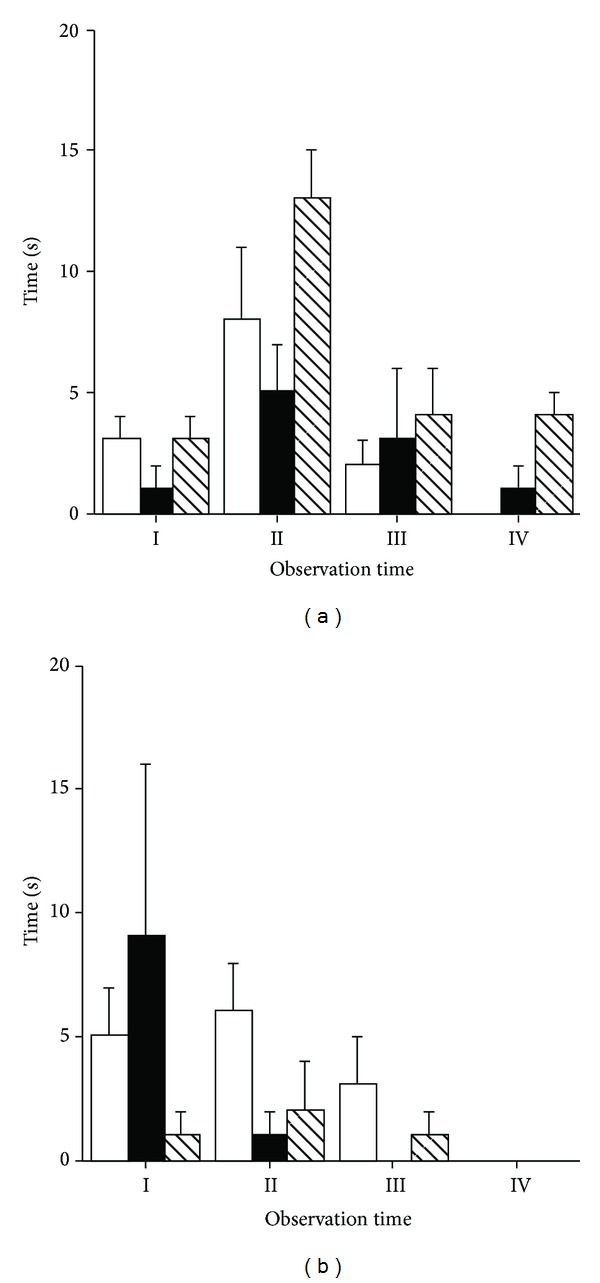
Effect of different types of physical exercise (race blank; maximal exercise shaded; daily exercise lined) on urinating (a) and defecating activity (b) of Standardbred horses housed in single stalls. Mares were examined at rest in their stalls (Time I), immediately after exercise (Time II), one (Time III), and two hours (Time V) after the completion of exercise. Values are expressed as means ± SE.

Eliminative behaviours (urinating and defecating) were significantly affected by time (*P* < 0.01 and *P* < 0.05, resp.) only. Defecating activity was significantly higher before the commencement of physical exercise (Time I; Figure 4(b)), while urinating activity was significantly higher during Time II compared to the other times of observation (Figure 4(a)).

**Figure 5:**
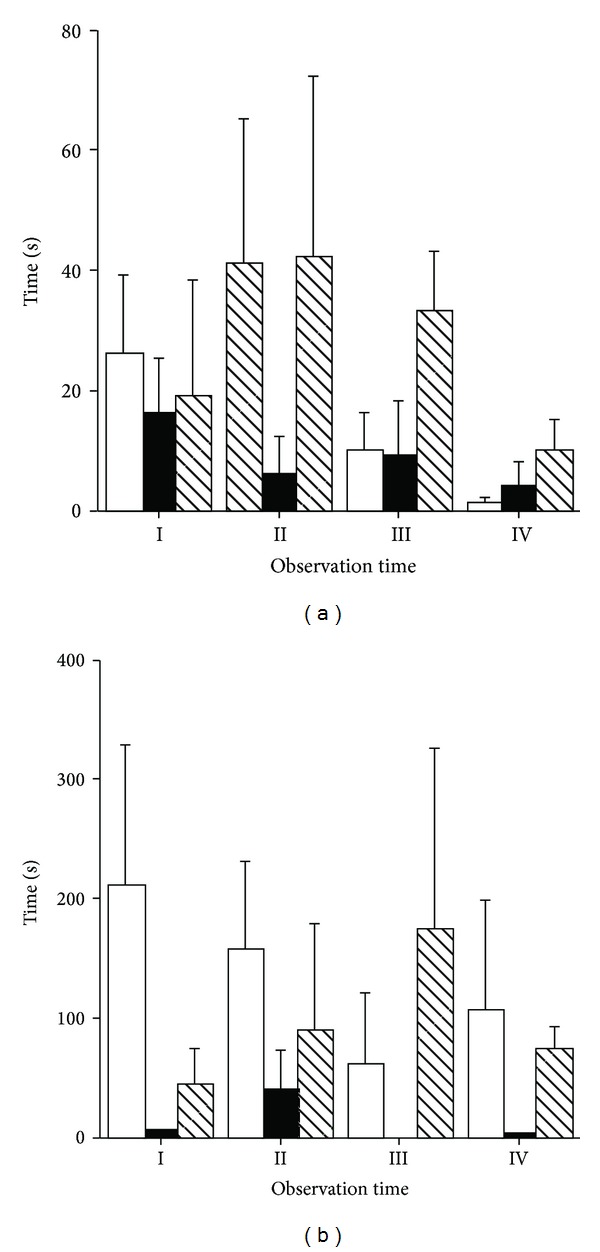
Effect of different types of physical exercise (race blank; maximal exercise shaded; daily exercise lined) on stereotypic behaviour (a) and exploitive activity (b) of Standardbred horses housed in single stalls. Mares were examined at rest in their stalls (Time I), immediately after exercise (Time II), one (Time III), and two hours (Time V) after the completion of exercise. Values are expressed as means ± SE.

Among the abnormal behaviours, crib-biting was the only one that was observed and only in two mares. No effect of physical exercise, time, and their interaction was observed for this stereotypic behaviour (Figure 5(a)) and explorative behaviours (Figure 5(b)).

## 4. Discussion 

The good health of the horses recruited in this study was substantiated by the rapid return of the physiological parameters (HR, RR, and RT) measured to baseline levels after the completion of the different physical exercises. As expected the increase in HR induced by DT was lower than that induced by ME and R. In particular, the HR increase was higher in horses that participated in the race event than those that performed ME, even though the horses covered the same distance at a similar speed, as the race not only represents a maximal exercise, but it also produces a stronger emotional response and mental stress [14]. Moreover, during a race event the presence of the audience might also have resulted in increased stress levels [15] and thus this emotional influence might explain the higher increase in HR observed in horses after the race. As expected, changes in RR were proportional to the intensity of the physical exercises. Horses rely primarily on sweating for heat loss, but the respiratory tract contributes to heat loss especially during exercise [16, 17]. Similarly, Miraglia et al. [18] found a positive correlation between the increase of RT and exercise intensity. Since it is well known that thermoregulation is impaired in poorly trained horses [17], we can conclude that our horses were in good health as their RT returned to preexercise levels within one hour from completion of the physical activity.

The duration of drinking activity was higher in horses after the race event than after ME and DT exercises; this behaviour was particularly evident during the first hour of observation, in accordance with Carson and Wood-Gush [19], who also reported an increase in the drinking activities soon after an intense exercise. In our study urinations occurred predominantly during the second hour of observation after exercise; a likely consequence of the increase in drinking activity was observed during the first hour after exercise, which indicates that the horses in this study quickly achieved a good state of hydration. Eating behaviour was particularly frequent during the first observation period after the race event and ME. Our findings are in agreement with those of Caanitz et al. [20], who proposed that the observed increase in time spent eating might have been a physiological consequence of the high energy expenditure during intense exercise. Increase in eating behaviour and in appetite has also been reported in trotters after a three-hour journey to restore the energy lost during transport [21]. However, our observations might also have been influenced by that fact that in this study horses were fasted for two hours prior to the race and ME events; therefore, the motivation to eat after these exercises could have been stronger.

Resting behaviour, such as lying down or standing while resting was less frequent in horses after R and ME than after DT. Usually horses only lie down only in a quiet environment [19]; however, in this study, when horses performed a race or ME, there were people, trainers, and drivers in their stable and therefore the horses might have been more alert, particularly after the race when the race track was usually very noisy [22]. Alternately, the horses might have been more active in their stalls in response to catecholamines released during the strenuous exercise [23].

There was no effect of type of exercise on explorative behaviour. Horses exhibit explorative behaviour to investigate new situations and environments [24], whereas horses well adapted to their environment and in good welfare present decreased explorative behaviour [25]; therefore, our findings suggest that in this study horses were well accustomed to their environment. It is important to note that explorative behaviour did not show any statistical difference between exercise types, but all horses showed explorative behaviours within the normal range and did not manifest any atypical posture. Therefore, in accordance with that reported by Fureix et al. [26] we can infer that the mares enrolled in this study did not suffer from depression.

Furthermore, our findings indicate that the incidence of abnormal behaviours in the horses recruited in this study was extremely low and that it was not affected by the type of physical exercise. One explanation for this observation is that our horses were quite young and therefore less prone to show stereotypies in agreement with Bachmann et al. [27]. Another explication could be that the horses were well managed and accustomed to the training regime. In fact, as suggested by Houpt and McDonnell [28], it is likely that good management also contributed to the low incidence of stereotypies. Although housing in single stalls and a stimuli poor environment is usually positively correlated with the development of abnormal behaviours [7], our data indicated that the horses were well adapted to their routinely management as they were trained on a daily basis, fed thrice, and handled many times a day. In addition, human activities (feeding, grooming, and cleaning) were conducted daily for 10 hours in the stable, suggesting that the horses were well accustomed to human presence and that they were handled and looked after properly. Finally, the presence of few crib-biting events could be more closely correlated with the feeding other than the training [7]. Therefore, in agreement with Werhahn et al. [11], our data suggest that a regular training regime does not increase the occurrence of stereotypies in Standardbred confined in single stalls.

## 5. Conclusion

In conclusion, after all physical exercises tested in this study, horses increased eating and drinking behaviour, whereas they decreased resting. In consequence, to guarantee a favourable physiological and behavioural restore, we would recommend that horses are offered food and fresh water in their boxes after cooling down. Overall, the assessment of horse behaviour after physical exercise by means of a focal ethogram represents a useful tool to monitor equine welfare. This could easily be adopted by the horse industry with the use of CCTV technology.

## References

[1] Werhahn H, Hessel EF, van den Weghe HFA (2012). Competition horses housed in single stalls (I): behavior and activity patterns during free exercise according to its configuration. *Journal of Equine Veterinary Science*.

[2] Padalino B, de Palo P, Tateo A, Centoducati P (2005). La gestione del cavallo trottatore: indagine in Puglia. *Ippologia*.

[3] Freire R, Buckley P, Cooper JJ (2009). Effects of different forms of exercise on post inhibitory rebound and unwanted behaviour in stabled horses. *Equine Veterinary Journal*.

[4] Ladewing J The other 23 hours of the day.

[5] VanDiereholock M, Vogel-vanVreeswijk T Revolutionary equine group housing system with automatic roughage feeding system moving in between a group: the effect of increasing from 3 to 6 feeding runs.

[6] Cooper JJ, Albentosa MJ (2005). Behavioural adaptation in the domestic horse: potential role of apparently abnormal responses including stereotypic behaviour. *Livestock Production Science*.

[7] McGreevy PD, Cripps PJ, French NP, Green LE, Nicol CJ (1995). Management factors associated with stereotypic and redirected behaviour in the thoroughbred horse. *Equine Veterinary Journal*.

[8] Cooper JJ, Mason GJ (1998). The identification of abnormal behaviour and behavioural problems in stabled horses and their relationship to horse welfare: a comparative review. *Equine Veterinary Journal Supplements*.

[9] Nicol C (1999). Understanding equine stereotypies. *Equine Veterinary Journal Supplements*.

[10] Pell SM, McGreevy PD (1999). A study of cortisol and beta-endorphin levels in stereotypic and normal Thoroughbreds. *Applied Animal Behaviour Science*.

[11] Werhahn H, Hessel EF, van den Weghe HFA (2012). Competition horses housed in single stalls (II): effects of free exercise on the behavior in the stable, the behavior during training, and the degree of stress. *Journal of Equine Veterinary Science*.

[12] Martin-Rosset W (1990). *L’alimentazione del cavallo*.

[13] Tateo A, Siniscalchi M, Padalino B, Dimatteo S, Centoducati P, Quaranta A (2008). Parametri ematochimici e fisiologici in cavalli trottatori sottoposti ad esercizio su bagnasciuga e in pista. *Ippologia*.

[14] Rietmann TR, Stuart AEA, Bernasconi P, Stauffacher M, Auer JA, Weishaupt MA (2004). Assessment of mental stress in warmblood horses: heart rate variability in comparison to heart rate and selected behavioural parameters. *Applied Animal Behaviour Science*.

[15] Becker-Birck M, Biau S, Ill N, Aurich J, Mostl E, Aurich C Heart rate, heart rate variability and cortisol release in the horse and its rider: different response to training and a public equestrian performance.

[16] Marlin D, Nankervis K (2002). *Equine Exercise Physiology*.

[17] Hodgson DR, Davis RE, McConaghy FF (1994). Thermoregulation in the horse in response to exercise. *The British Veterinary Journal*.

[18] Miraglia N, Bergero D, Gagliardi D (2000). *Il cavallo atleta*.

[19] Carson K, Wood-Gush DGM (1983). Equine behaviour: II. A review of the literature on feeding, eliminative and resting behaviour. *Applied Animal Ethology*.

[20] Caanitz H, O’Leary L, Houpt K, Petersson K, Hintz H (1991). Effect of exercise on equine behavior. *Applied Animal Behaviour Science*.

[21] Tateo A, Padalino B, Boccaccio M, Maggiolino A, Centoducati P (2012). Transport stress in horses: effects of two different distances. *Journal of Veterinary Behavior*.

[22] Kiley-Worthington M (1990). The behavior of horses in relation to management and training—towards ethologically sound environments. *Journal of Equine Veterinary Science*.

[23] Hagan JJ, Bohus B (1983). The effects of endorphins on cardiac responses during an emotional stress. *Physiology and Behavior*.

[24] McGreevy P (2008). *Equine Behaviour: A Guide for Veterinarians and Equine Scientists*.

[25] Mench JA, Mason GJ (1997). *Animal Welfare*.

[26] Fureix C, Jego P, Henry S, Lansade L, Hausberger M (2012). Toward an ethological animal model of depression? A study on horses. *PLoS ONE*.

[27] Bachmann I, Audigé L, Stauffacher M (2003). Risk factors associated with behavioural disorders of crib-biting, weaving and box-walking in Swiss horses. *Equine Veterinary Journal*.

[28] Houpt KA, McDonnell SM (1993). Equine stereotypes. *Compendium on Continuing Education for the Practising Veterinarian*.

